# A competing risk joint model for dealing with different types of missing data in an intervention trial in prodromal Alzheimer’s disease

**DOI:** 10.1186/s13195-021-00801-y

**Published:** 2021-03-22

**Authors:** Floor M. van Oudenhoven, Sophie H. N. Swinkels, Hilkka Soininen, Miia Kivipelto, Tobias Hartmann, Dimitris Rizopoulos, on behalf of the LipiDiDiet clinical study group, Tobias Hartmann, Hilkka Soininen, Miia Kivipelto, Alina Solomon, Pieter Jelle Visser

**Affiliations:** 1 Department of Biostatistics, Erasmus Medical Center, PO Box 2040, 3000 Rotterdam, CA the Netherlands; 2 Danone Nutricia Research, Uppsalalaan 12, 3584 CT Utrecht, The Netherlands; 3 Department of Neurology, Institute of Clinical Medicine, University of Eastern Finland, PO Box 1627, 70211 Kuopio, Finland; 4 Neurocenter, Department of Neurology, Kuopio University Hospital, PO Box 100, 70029 Kuopio, Finland; 5 Division of Clinical Geriatrics, Department of Neurobiology, Care Sciences and Society, Karolinska Institute, 14157 Huddinge, Sweden; 6 Clinical Trials Unit, Theme Aging, Karolinska University Hospital, 14152 Huddinge, Sweden; 7 Institute of Public Health and Clinical Nutrition, University of Eastern Finland, P.O. Box 1627, 70211 Kuopio, Finland; 8 Ageing Epidemiology Research Unit, School of Public Health, Imperial College London, St Dunstan’s Road, London, UK; 9 Deutsches Institut für Demenz Prävention (DIDP), Medical Faculty, Saarland University, Kirrbergerstraße, 66421 Homburg, Germany; 10 Department of Experimental Neurology, Saarland University, Kirrbergerstraße, 66421 Homburg, Germany

**Keywords:** Alzheimer’s disease, Prodromal, Joint model, Fortasyn, Randomized controlled trial, Dropout, Dietary intervention

## Abstract

**Background:**

Missing data can complicate the interpretability of a clinical trial, especially if the proportion is substantial and if there are different, potentially outcome-dependent causes.

**Methods:**

We aimed to obtain unbiased estimates, in the presence of a high level of missing data, for the intervention effects in a prodromal Alzheimer’s disease trial: the LipiDiDiet study. We used a competing risk joint model that can simultaneously model each patient’s longitudinal outcome trajectory in combination with the timing and type of missingness.

**Results:**

Using the competing risk joint model, we were able to provide unbiased estimates of the intervention effects in the presence of the different types of missingness. For the LipiDiDiet study, the intervention effects remained statistically significant after this correction for the timing and type of missingness.

**Conclusion:**

Missing data is a common problem in (Alzheimer) clinical trials. It is important to realize that statistical techniques make specific assumptions about the missing data mechanisms. When there are different missing data sources, a competing risk joint model is a powerful method because it can explicitly model the association between the longitudinal data and each type of missingness.

**Trial registration:**

Dutch Trial Register, NTR1705. Registered on 9 March 2009

**Supplementary information:**

**Supplementary information** accompanies this paper at 10.1186/s13195-021-00801-y.

## Background

The course of Alzheimer’s disease (AD) is characterized by a process of neurodegenerative changes, gradually leading to subtle cognitive decline several years before the diagnosis of AD dementia can be made [1]. As such, clinical trials in AD studying the efficacy on disease progression typically require long follow-up periods [2]. A frequent problem associated with follow-up studies is missing data, especially in the case of long-term follow-up. Missing data may result from subjects dropping out of a trial, for instance, when they move away or lose motivation to participate. If subjects who terminate the trial early are systematically different from completers, the resulting missing data pose challenges on the statistical analysis.

This paper focuses on the data of a randomized controlled trial in individuals with prodromal AD, the LipiDiDiet trial [3, 4]. The trial had an initial 24-month intervention period, with the trial design allowing subjects to continue for a maximum of 72 months of randomized, controlled, double-blind, parallel-group intervention. This paper addresses the first 36 months of intervention, which currently is the maximum reported intervention period. The trial’s objective was to assess the effect of medical nutrition (Souvenaid) on cognition and related measures. The active component of Souvenaid is Fortasyn Connect, a specific combination of nutrients that reduces AD-linked brain pathologies in a neuroprotective manner [5–11]. Previous clinical studies showed benefits on memory and functional connectivity in patients with mild and moderate AD [12–14].

In the LipiDiDiet trial, significant benefits of the intervention over the placebo control arm were observed for the Neuropsychological Test Battery (NTB) score, NTB 5-item composite, and NTB memory domain, the Clinical Dementia Rating Sum of Boxes (CDR-SB), which is a measure of cognition and function in real life, as well as on multiple measures of brain atrophy [3, 4].

As typical for long-term clinical trials, the LipiDiDiet trial had missing data. In particular, about 25% of the randomized subjects had data eligible for efficacy analysis at the last 36-month time point. One of the reasons for these missing data was the exclusion (i.e., censoring) of data collected after the start of open-label medication given to subjects who progressed to dementia during the trial. That is, although subjects went to AD medication or open-label Souvenaid intervention, instead of double-blind placebo-controlled active intervention in the absence of pharmaceutical AD medication, they were allowed to remain in the trial, and data were continued to be recorded. However, these data were -prespecified to be- excluded from the main analyses. The reasons for excluding these data include that AD drugs (potent neurotransmitter level modulators) are likely to affect the study outcomes and that the switch to the open-label intervention terminates the double-blind phase of the trial for these subjects. Note that although data of some subjects on open-label medication were still collected and therefore not missing, throughout this paper, we refer to these as missing data since these data were not used in the statistical analysis.

Apart from excluding data after the use of open-label medication, also other reasons contributed to the pool of missing data, in this case, because data could not be recorded. These include not opting in after the first 24 months (*n* = 64), adverse events (*n* = 18), withdrawal of informed consent (*n* = 22), protocol deviations (*n* = 3), other reasons (*n* = 37), or lost to follow-up (*n* = 4).

Therefore, throughout this paper, we distinguish two groups of subjects, each with a different type of missing data. The first group, i.e., missing group 1, refers to subjects with missing data due to the exclusion (i.e., censoring) of data collected after the start of open-label medication. The second group, i.e., missing group 2, refers to subjects who dropped out of the trial. We also identify a third group, the completers. This group refers to subjects who remained in the trial for 36 months without dropping out or censoring of data.

The longitudinal measures that were collected in the LipiDiDiet trial include an NTB 5-item composite *Z* score, composite *Z* scores for NTB memory domain, NTB executive function domain, and NTB total based on 16 items, CDR-SB, hippocampal, ventricular, and whole-brain atrophy based on MRI [3, 4]. Since a worsening of cognition is among the criteria for AD dementia diagnosis [15], one could expect a (strong) correlation between the longitudinal outcomes on cognition and related measures and progression to dementia. Therefore, whether or not a subject has missing data due to the exclusion of data after using open-label medication following progression to dementia should typically be related to their (missing) longitudinal measurements. For this reason, the missingness is possibly informative. In this case, the traditional statistical methods for analyzing longitudinal data, i.e., mixed models, are subject to bias. Instead, a method that takes into account the missing data mechanism needs to be used. Previous data analysis included a sensitivity analysis using a joint model for longitudinal and survival data [16–18]. Such an analysis can take into account the possible informative character of the missing data by simultaneously modeling the longitudinal data and the event and timing of missingness [19, 20]. However, it is reasonable to assume that the dependence between the missingness and the longitudinal measurements might also have been dependent on the reason for missingness. That is, we can expect different (missing) longitudinal trajectories for subjects whose data collected after the start of open-label medication use were censored than for subjects who dropped out of the trial. These two types of (missing) longitudinal trajectories might differ from the trajectories of the subjects who completed the trial without dropout or censoring of data. In this paper, we use a competing risk joint model that can deal with and distinguish between the completers and the two different types of missing data. For information on competing risk joint models, see, for example, [21, 22]. Within the context of AD, these models have been addressed previously to model the competing risks of dementia and death [23, 24]. Using the competing risk joint model, we aim to obtain unbiased estimates of the intervention effects in the presence of the different types of missingness. Additionally, we compare the results based on mixed models, standard joint models, and the competing risk joint models, i.e., three methods that deal differently with the missing data.

## Methods

### Study design and subjects

The LipiDiDiet trial is a randomized, controlled, double-blind, parallel-group, multicenter trial done primarily in memory clinics at 11 study sites in Finland, Germany, The Netherlands, and Sweden. Following the 24-month intervention [3], subjects could continue for 72 months of randomized, controlled, double-blind, parallel-group intervention. In this paper, we report over 36 months of intervention. The LipiDiDiet trial investigated the effects of Fortasyn Connect on cognition and related measures in individuals with prodromal AD. These included an NTB 5-item composite *Z* score, and composite *Z* scores for NTB memory domain, NTB executive function domain, and NTB total based on 16 items; CDR-SB; (three-dimensional T1-weighted) anatomical scans of the total hippocampal, whole brain, and ventricular volumes (cm^3^); and time to dementia diagnosis. For the outcome measures based on the NTB, composite Z scores were calculated standardized to the baseline mean and standard deviation (SD) based on the modified intention-to-treat population in the LipiDiDiet main papers [3, 4].

**Fig. 1:**
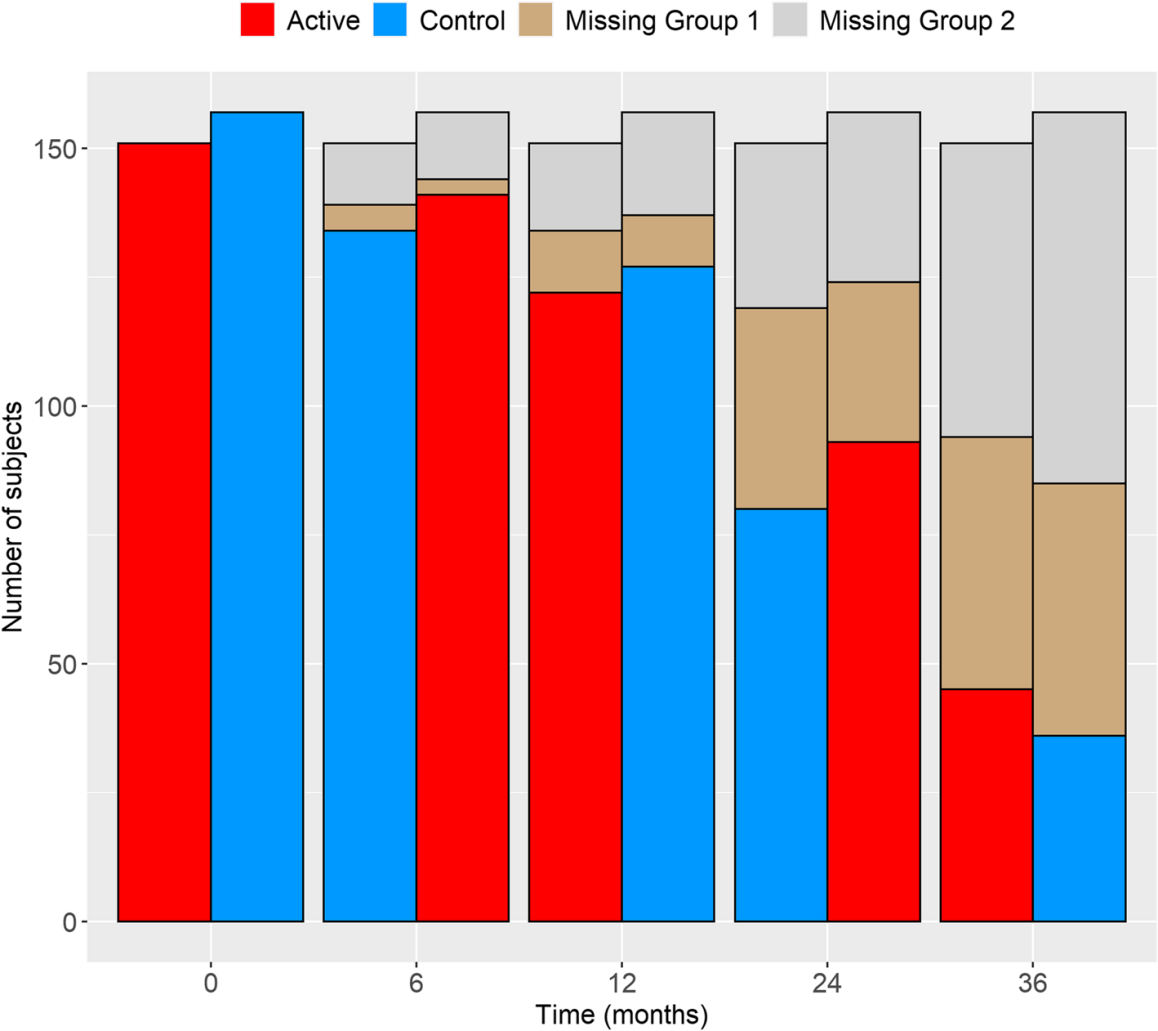
Number of subjects with measurements eligible for efficacy analysis of the primary endpoint

The diagnosis of dementia was made based on the criteria defined by DSM-IV, the National Institute of Neurological and Communicative Disorders and Stroke, and the AD and Related Disorders Association criteria for AD. Subjects were measured at baseline, and around 12, 24, and 36 months, with an additional visit around 6 months for the NTB *Z* scores. Figure 1 shows the number of subjects with available measurements for the primary endpoint at different time points in the competing risk joint model and the corresponding types of missingness. As can be seen, 45 (30%) and 36 (23%) subjects in the active and control groups, respectively, had data at the 36-month time point eligible for efficacy analysis. For more information, regarding the LipiDiDiet trial and the main paper reporting the 36-month intervention period results, we refer to the original clinical trial publications [3, 4].

### Investigating the missing data patterns

In this paper, we use a statistical method that distinguishes between three types of subjects: (1) subjects who completed the 36-month intervention period (completers), (2) subjects whose data collected after the start of open-label medication use were censored (missing group 1), and (3) subjects whose data were not recorded once they dropped out of the trial (missing group 2). To get insight into how different these three types of subjects are, we plotted the mean of their observed trajectories until the moment of missingness for NTB 5-item composite and CDR-SB.

### Statistical analyses

We simultaneously modeled the risks of missingness due to open-label medication use (missing group 1) and dropout (missing group 2), and the longitudinal trajectories, using joint modeling for longitudinal and survival data. Using a joint model might have advantages compared to using a mixed model in terms of how it deals with the missing data. According to Rubin’s taxonomy [25], we can distinguish three different mechanisms for missing data. When missingness is unrelated to the data, the mechanism is called missing completely at random (MCAR). When missingness depends only on the observed data (and covariates) but not on the unobserved data, the mechanism is termed missing at random (MAR). A mechanism where missingness depends on the unobserved data, perhaps in addition to the observed data, is missing not at random (MNAR). Note that in this paper, with (missing) data, we are referring to (missing) outcome data and not (missing) covariate data. The mixed model can accommodate both data with an MCAR and MAR mechanism. Joint models correspond to an MNAR missing data mechanism because they jointly model the longitudinal and the missingness process. For example, a joint model (with the risk of missingness as an event) assumes that, given the observed data and covariates, whether or not data of a subject is missing, and the timing of missingness might contain some additional information and should be taken into account. The competing risk joint model can thereby distinguish between two types of MNAR. For example, as done in this paper, in addition to using the information on whether or not data of a subject is missing and the timing of missingness, it also takes into account the reason for missingness, allowing each type of missingness to have a (different) MNAR characterization.

In the joint model, we simultaneously modeled the following three sub-models: (i) a longitudinal mixed model aiming to describe the patient-specific longitudinal trajectories, (ii) a Cox proportional hazard model for the risk of open-label medication use as an event, and (iii) a Cox proportional hazard model for the risk of dropout as a competing event.

For the longitudinal outcome of interest, suppose *y*_*i*_(*t*) were the observations for the *i*th subject at time points *t*_*ij*_ (*j =* 1,…, *n*_*i*_). Note that both the number of measurements (*n*_*i*_) and the timing of measurements (*t*_*ij*_) could vary between subjects. We used the following joint model:

$$ {y}_(i)(t)={m}_(i)(t)+{\varepsilon}_(i)(t), $$

$$ {m}_i(t)={\beta}_0+{\beta}_1t+{\beta}_2\ {\mathrm{intervention}}_i+{\beta}_3\ {\mathrm{intervention}}_i\ x\ t+{\beta}_4b{\mathrm{MMSE}}_i+{\beta}_5{\mathrm{site}}_i+{b}_{i0}+{b}_{i1}t+{b}_{i2}{t}^2, $$

$$ {h}_{iGr1}(t)={h}_{oGr1}(t)\exp \left\{{\gamma}_1{\mathrm{intervention}}_i+{\gamma}_2b{\mathrm{MMSE}}_i+{\alpha}_{Gr1}{m}_i(t)\right\}, $$

$$ {h}_{iGr2}(t)={h}_{oGr2}(t)\exp \left\{{\gamma}_1{\mathrm{intervention}}_i+{\gamma}_2b{\mathrm{MMSE}}_i+{\alpha}_{Gr2}{m}_i(t)\right\}. $$

In the longitudinal sub-model, we used linear time trends (*β*_1_) for the longitudinal outcomes. To model the effect of Fortasyn Connect, we included both a main effect (*β*_2_) and a linear interaction of the intervention effect by time (*β*_3_). In this way, *β*_2_ denotes the difference between the intervention groups at baseline, while the interaction effect describes the intervention effect over time. Further, we adjusted for the effect of baseline Mini-Mental State Examination (MMSE) (*β*_4_) and site (*β*_5_). To allow subjects to have different baseline levels and time trends, we included subject-specific random intercepts (*b*_*i*0_) and slopes (*b*_*i*1_). Additionally, quadratic random effects (*b*_*i*2_) were included if they improved the fit of the model. In the Cox model, we included intervention (*γ*_1_) and baseline MMSE (*γ*_*2*_) as time-independent effects and one of the longitudinal measures as a time-dependent effect. Further, *h*_*iGr*1_ and *h*_*iGr*2_ denote the risks of being in missing group 1 and missing group 2 for the *i*th subject. The quantity ε_*i*_(*t*) denotes the measurement error, for which we assumed ε_*i*_(*t*) ∼ *N* (0*, σ*^2^). The quantities *h*_*0Gr*1_ and *h*_*0Gr*2_ denote the baseline hazards for being in missing group 1 and missing group 2. The parameters *α*_*Gr*1_ and α_*Gr*2_ measure the strength of the association between the longitudinal outcome and the risk of the corresponding event. Specifically, the quantities exp(*α*_*Gr*1_) and exp(*α*_*Gr*2_) denote the hazard ratios for the competing event at time *t* for a unit increase in the longitudinal trajectory at the same time point.

Within the joint modeling framework, different possibilities exist to model the association between the longitudinal outcome and the risk of an event. For example, next to using the value of the longitudinal outcome, the rate of change (i.e., slope) could be used to model the relationship with the risk of an event. For more information about the different possibilities for the association structure, see [18, 26].

We also fitted a separate longitudinal mixed model and a standard joint model with the same covariate structure as in the competing risk joint model. In the standard joint model, we used missingness due to any possible reasons as a composite event. All the statistical analyses in this paper were performed with the statistical software package R, using R-package JM [27]. For an example of fitting a competing risk joint model using the R-package JM, including R code and an example dataset, we refer to [18].

## Results

### Investigating the missing data patterns

**Fig. 2:**
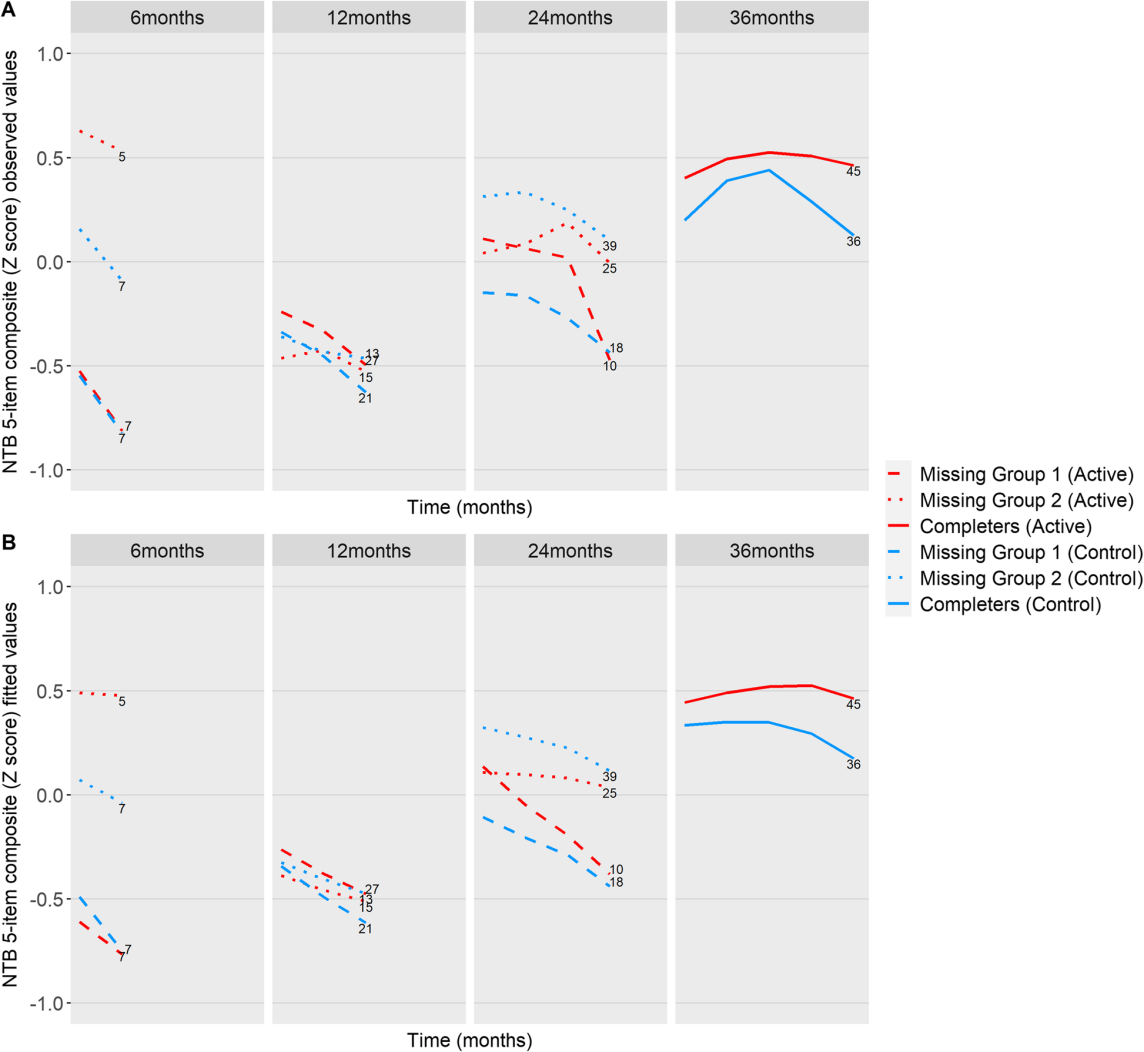
**a** The mean observed trajectories up to the last available measurement per group for NTB 5-item composite. **b** The mean fitted trajectories up to the last available measurement per group for NTB 5-item composite. The numbers denote the number of subjects per group. A higher NTB 5-item score indicates better performance

**Fig. 3:**
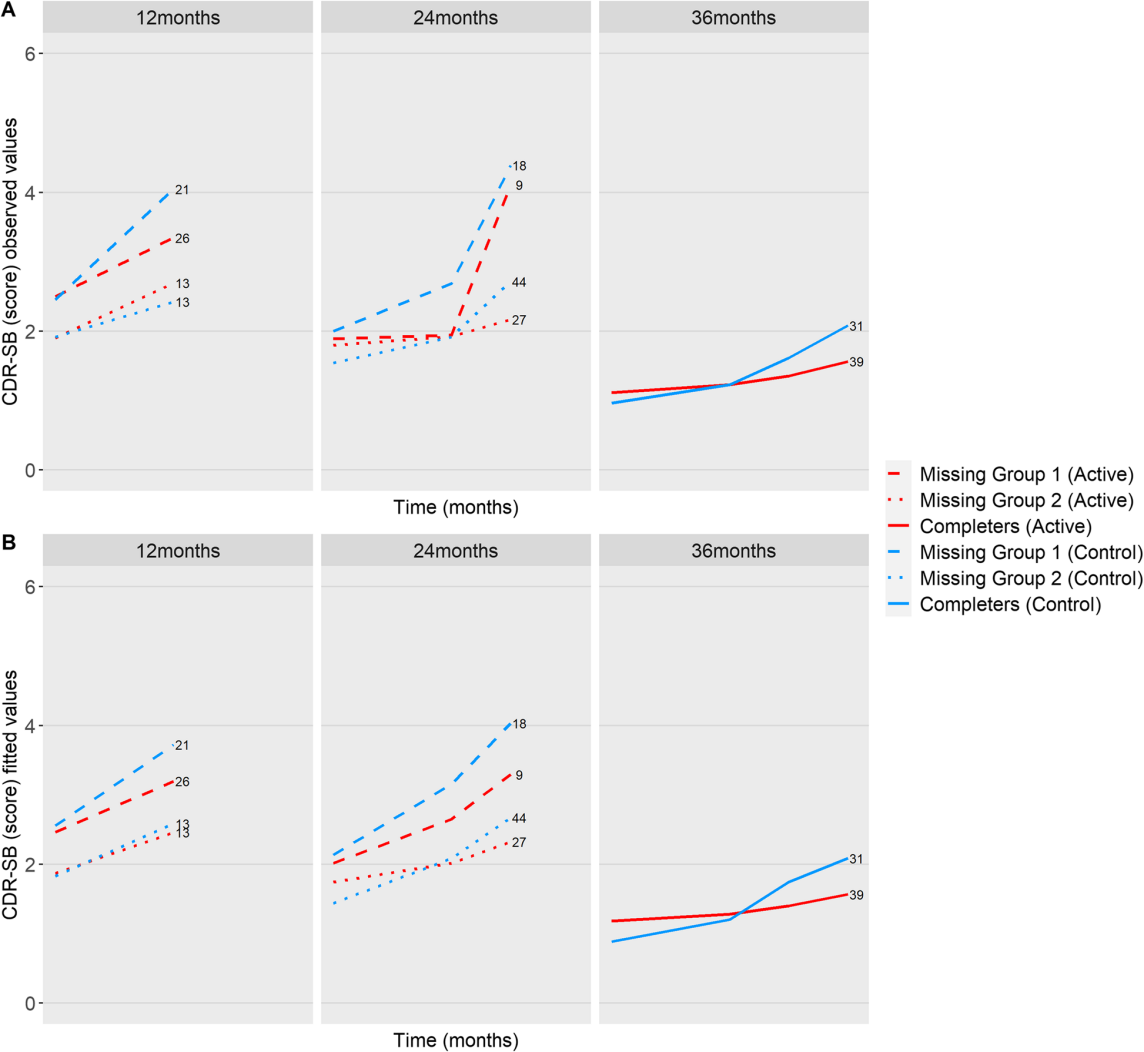
**a** The mean observed trajectories up to the last available measurement per group for CDR-SB. **b** The mean fitted trajectories up to the last available measurement per group for CDR-SB. The numbers denote the number of subjects per scenario of dropout. A higher CDR-SB score indicates worse performance

Figures 2a and 3a show the mean of the observed trajectories until the moment of missingness respectively for NTB 5-item composite and CDR-SB. For example, the 6-month panel shows the mean of the observed trajectories until 6 months for all subjects who only have data for up to 6 months. The 12-month panel then includes all subjects whose last data were recorded at month 12, etc. Although we cannot be sure about the missing trajectories, plotting the available information provides insight into the differential patterns. First, we observe a general pattern that the higher the baseline cognitive performance according to the NTB 5-item composite, the longer the subjects remained in the trial. Second, subjects with missingness due to open-label medication use seem to have different longitudinal trajectories than subjects who dropped out. A steeper decrease in NTB 5-item composite and a steeper increase in CDR-SB reflect a faster loss of cognitive performance and cognitive-functional performance.

### Statistical analyses

This paper’s results can differ from the LipiDiDiet main paper presenting the 36-month results [4] since different modeling choices are made. The main difference is that in this paper, we used the competing risk joint model, which can take into account the possible informative character of the missing data and thereby also distinguishes between the reasons for missingness as defined here. Furthermore, the main paper’s approach is a mixed model that includes the outcome baseline value as a covariate and models the change from baseline as the response variable, according to a prespecified statistical analysis plan. However, modeling the outcome baseline values as part of the trajectory is preferred when using a joint model, as it maximizes the amount of information used to estimate the association between the longitudinal data and the survival data. Therefore, we included the baseline values in the longitudinal trajectory and modeled the value as the response variable.

**Fig. 4:**
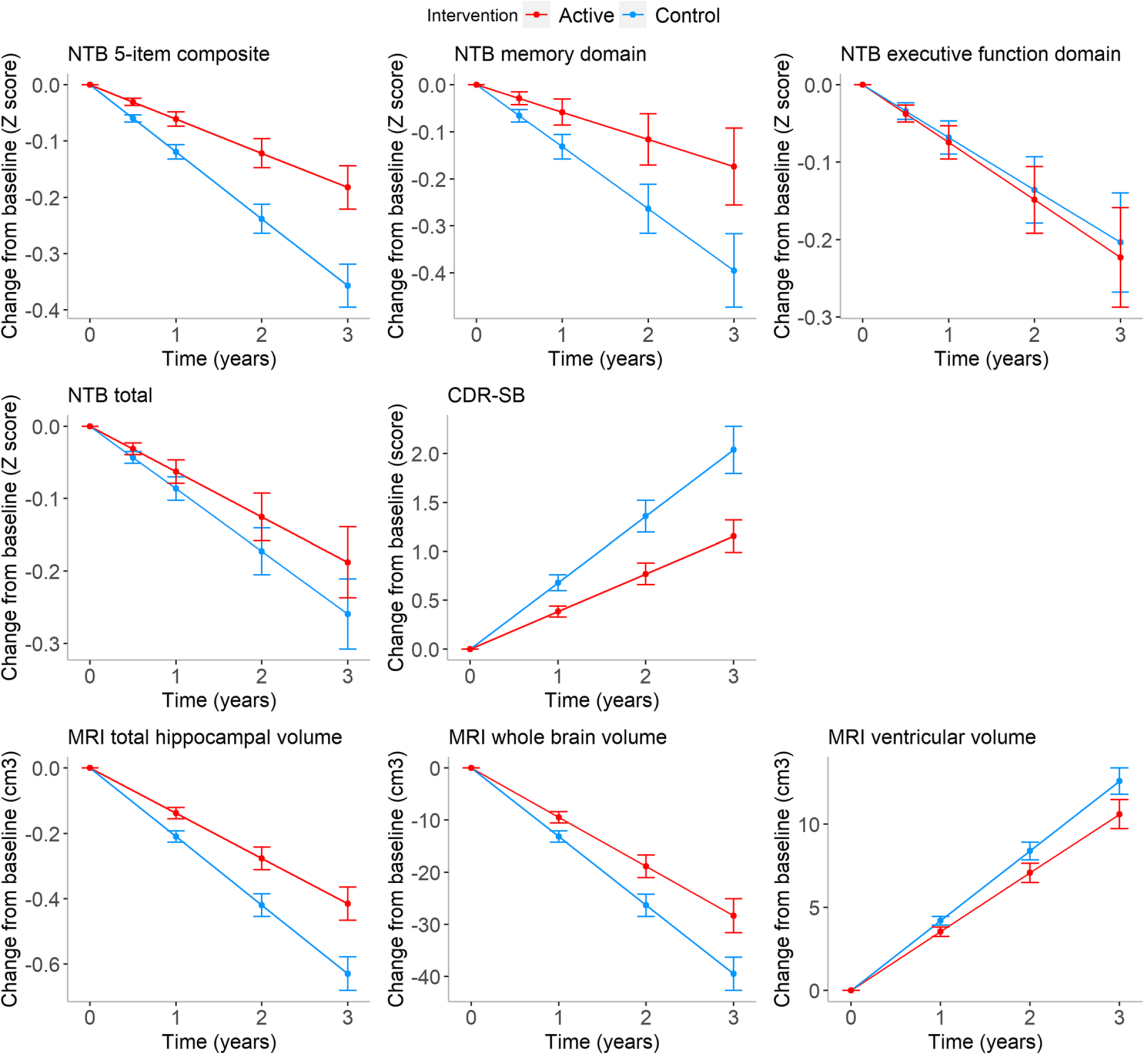
Estimated mean change from baseline based on the competing risk joint model for each outcome. Error bars are ± SE. Except for CDR-SB and MRI ventricular volume, higher scores indicate better performance

Supplementary Table 1 shows the baseline characteristics for missing group 1, missing group 2, and the completers. Supplementary Table 2 shows the number of subjects (*n*), the number of total observations (*N*), and the number of the competing events in the competing risk joint model for each longitudinal outcome in the control and active groups. Figure 4 presents the estimated mean change from baseline as estimated from the competing risk joint model. As can be seen, trajectories worsened over time. We also plotted the mean of the fitted trajectories, i.e., the estimated means for each type of missing data until the moment of missingness respectively for NTB 5-item composite (Fig. 2b) and CDR-SB (Fig. 3b).

Table 1 (A) shows the longitudinal sub-model results of the competing risk joint model for each longitudinal outcome. Note that for NTB 5-item composite, NTB executive function domain, NTB total, MRI total hippocampal volume, MRI whole brain volume, and MRI ventricular volume, we included random quadratic effects as it improved the fit of the model. The parameter *β*_3_ describes the intervention effect on the corresponding longitudinal outcome over time and denotes our effect size of interest. For NTB 5-item composite and NTB memory domain, we observe significant intervention effects, with the average decrease being respectively 0.058 (95% CI 0.023 to 0.094) and 0.074 (95% CI 0.003 to 0.145) per year less in the active group than in the control group. For NTB executive function domain and NTB total, we observe −0.006 (95% CI −0.064 to 0.052) and 0.024 (95% CI −0.019 to 0.067) less reduction per year in the active group than in the control group, respectively, although not being statistically significant. We observe significantly less worsening in CDR-SB in the active group than in the control group, with an estimated difference of −0.295 (95% CI −0.480 to −0.109) per year. Additionally, we observe significant intervention effects for MRI total hippocampal volume and MRI whole brain volume, with respectively 0.071 (95% CI 0.028 to 0.115) and 3.719 (95% CI 0.829 to 6.609) less reduction per year in the active group than in the control group. We observe an estimated yearly difference of −0.659 (95% CI − 1.426 to 0.108) for MRI ventricular volume, although not being statistically significant.

**Table 1:** Competing risk joint model results for the (A) longitudinal and (B) survival sub-model

**A. Longitudinal sub-model**				
Longitudinal outcome	*β*_3_^*^ (95% CI)	pVal	Intervention effect over 36 months (95% CI)	
NTB 5-item composite (*Z* score)	0.058 (0.023 to 0.094)	0.001	0.175 (0.069 to 0.281)	
NTB memory domain (*Z* score)	0.074 (0.003 to 0.145)	0.042	0.221 (0.008 to 0.434)	
NTB executive function domain (*Z* score)	−0.006 (−0.064 to 0.052)	0.828	−0.019 (−0.193 to 0.155)	
NTB total (*Z* score)	0.024 (−0.019 to 0.067)	0.278	0.071 (−0.058 to 0.200)	
CDR-SB (score)	−0.295 (−0.480 to −0.109)	0.002	−0.884 (−1.440 to − 0.328)	
MRI total hippocampal volume (cm^3^)	0.071 (0.028 to 0.115)	0.001	0.214 (0.084 to 0.344)	
MRI whole brain volume (cm^3^)	3.719 (0.829 to 6.609)	0.012	11.157 (2.487 to 19.827)	
MRI ventricular volume (cm^3^)	−0.659 (−1.426 to 0.108)	0.092	−1.978 (−4.279 to 0.324)	
**B. Survival sub-model**				
Longitudinal outcome	Missing group 1: open-label medication use		Missing group 2: dropout	
	*α*_*Gr*1_	HR (95% CI)	*α*_*Gr*2_	HR (95% CI)
NTB 5-item composite (*Z* score)	−1.483	0.227 (0.164 to 0.315)	1.266	3.548 (2.332 to 5.400)
NTB memory domain (*Z* score)	−1.208	0.299 (0.216 to 0.414)	1.094	2.987 (2.001 to 4.459)
NTB executive function domain (*Z* score)	−0.711	0.491 (0.351 to 0.687)	0.444	1.559 (1.000 to 2.429)
NTB total (*Z* score)	−1.661	0.190 (0.123 to 0.294)	1.373	3.947 (2.250 to 6.923)
CDR-SB (score)	0.497	1.644 (1.466 to 1.842)	−0.513	0.599 (0.501 to 0.715)
MRI total hippocampal volume (cm^3^)	−0.530	0.588 (0.477 to 0.726)	0.532	1.703 (1.299 to 2.232)
MRI whole brain volume (cm^3^)	−0.002	0.998 (0.995 to 1.001)	0.000	1.000 (0.996 to 1.004)
MRI ventricular volume (cm^3^)	0.013	1.013 (1.006 to 1.021)	−0.010	0.990 (0.980 to 1.000)

*β*_3_ denotes the yearly intervention effect. Except for CDR-SB and MRI ventricular volume, higher scores indicate better performance

**Fig. 5:**
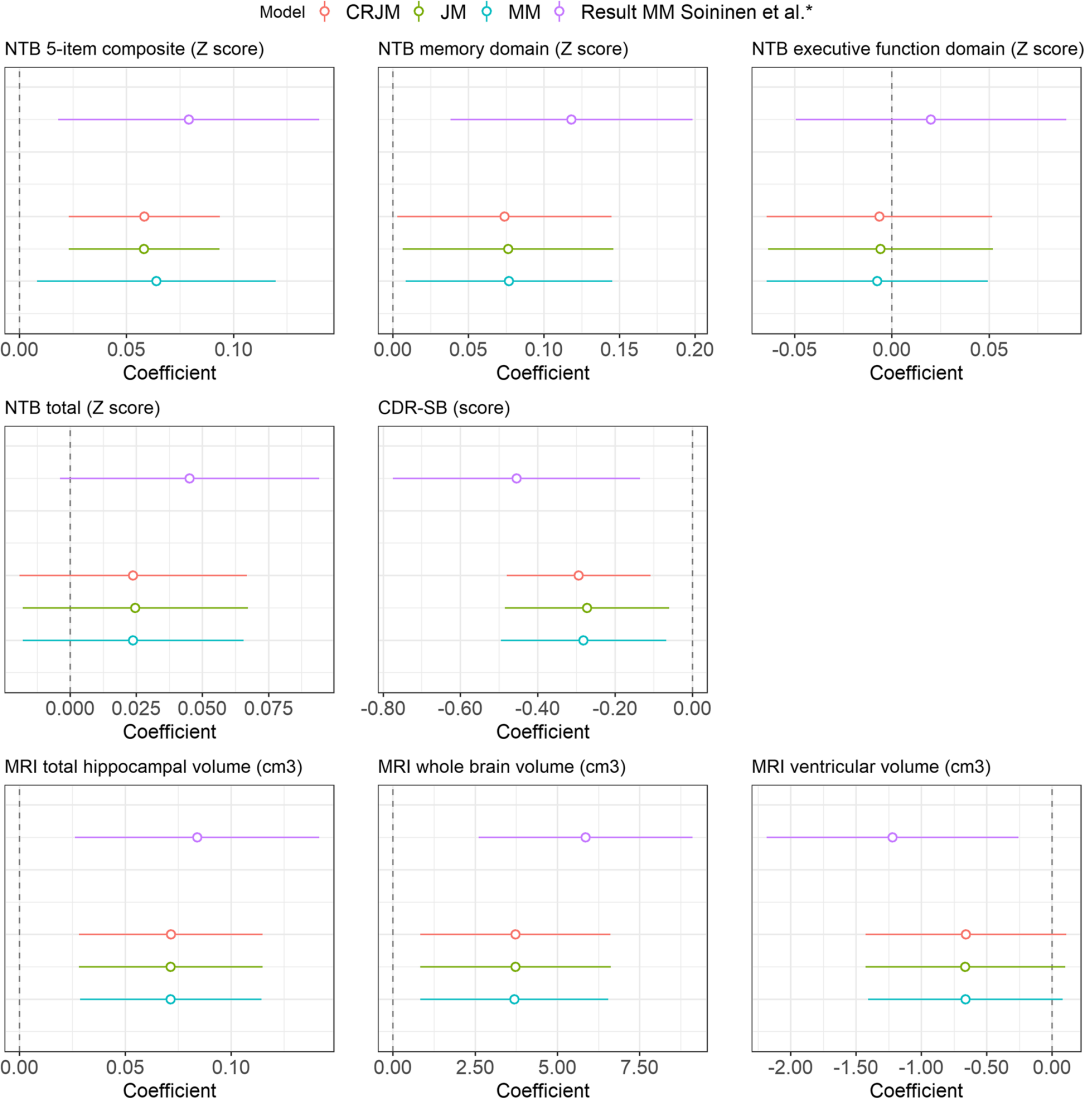
Estimated parameters for the yearly intervention effect for each outcome. Intervention effects are based on the competing risk joint model (CRJM), joint model (JM), and mixed model (MM). Error bars are the corresponding 95% CI. *In Soininen et al., the resulting estimated changes over the 36-month period were presented

Figure 5 compares the intervention effect estimates and corresponding 95% confidence intervals for each longitudinal outcome obtained from the competing risk joint model, the standard joint model, and the separate linear mixed model. This figure also includes the results as presented in the LipiDiDiet main paper [4]. We observe that the results for the intervention effects are comparable for the three types of models. For the interested reader, we refer to Supplementary Figure 1, which provides more insight into the differences between the models. This figure shows that, although the intervention effect estimates are comparable, the models differ in how they deal with the three different types of subjects. Also, we conducted two small data manipulations solely to illustrate the potential bias that could be introduced by ignoring the missingness and the gain in efficiency that can be achieved by modeling the different types of missingness. For both data manipulations, we used the NTB 5-item composite outcome. For the first type of data manipulation, we artificially created extra MNAR missingness in the control group for those subjects who decreased the most in cognitive performance. For the second type of data manipulation, we artificially created two different types of MNAR missingness. In particular, we created extra MNAR missingness in the control group for those subjects who decreased the most in cognitive performance and extra MNAR missingness in the treatment group for those subjects who improved the most in cognitive performance. As can be seen, in these situations, the mixed model, joint model, and competing risk joint model yield different results (see Supplementary Tables 4 and 5).

To enable evaluation of the model goodness of fit, we also added plots of the residuals versus the fitted values for each outcome to the Supplementary Material (Supplementary Figure 2). Overall, we conclude that the fit is satisfactory.

Table 1 (B) also shows the survival sub-model results of the competing risk joint model for each longitudinal outcome. The sign of the *α*-coefficient indicates the direction of the association. For the NTB composite scores, MRI total hippocampal, and whole brain volume, the sign of the α-coefficient is negative for missing group 1, indicating that a higher value is found to be associated with a lower risk of being in missing group 1 (i.e., open-label medication use). For example, a unit increase in NTB 5-item composite score is estimated to increase the risk of being in missing group 1 by 0.227-fold (95% CI 0.164 to 0.315), which means an estimated risk reduction of 77.3%. For CDR-SB and MRI ventricular volume, the sign of the α-coefficient is positive for missing group 1, indicating that a higher value is found to be associated with a higher risk of being in missing group 1. For instance, a unit increase in CDR-SB is estimated to increase the risk of being in missing group 1 by 1.644-fold (95% CI 1.466 to 1.842), which means an increase of 64.4%. Note that associations and hazard ratios corresponding to one unit increase in the different types of longitudinal outcomes are not directly comparable as the longitudinal outcomes are measured on different scales.

Using the rate of change (i.e., slope) to model the association between the longitudinal outcome and the risk of an event was found to give similar results for the intervention effects (see Supplementary Table 3).

## Discussion

In this paper, we aimed to obtain unbiased estimates for the intervention effect on the longitudinal outcomes in the presence of missing data. One of the reasons for the missing data was the exclusion of data collected after the start of open-label medication given to subjects who progressed to dementia during the trial, but also other reasons contributed to the pool of missing data. We applied a statistical method that simultaneously models the longitudinal trajectories and the event and timing of missingness and thereby distinguishes between the different types of missing data. We believe that the so-called competing risk joint model is an adequate way to deal with this type of data. We found significant intervention effects for three longitudinal outcomes in the neuropsychological domain (NTB 5-item composite, NTB memory domain, and CDR-SB) and two longitudinal MRI brain volume measures (hippocampal and whole brain volume).

Apart from using the longitudinal outcome’s value to model the association with the risk of an event, we also tried using the rate of change (i.e., slope). However, results for the intervention effects were found to be very similar. One explanation might be that the study population is relatively homogeneous at baseline, with all subjects being at a similar (i.e., prodromal) disease stage. In this situation, the difference between subjects in the post-baseline value is highly correlated with differences between subjects in slopes. In a population with different disease stages and thus with substantial differences in baseline levels, results might be more sensitive to the choice of the association structure.

Similar results regarding the intervention effect were obtained based on the competing risk joint model, the joint model, and the mixed model. These three types of models make different assumptions about the missing data mechanism. Mixed models can accommodate data that is MAR. When the missing data is MAR, this means that the missing data can be predicted based on the observed data and covariates. The standard joint model used in this paper takes into account that missing data might be MNAR, and the competing risk joint model allows the pattern of dependence between the probability of missingness and the unobserved data to be different for the different types of missingness. The fact that the obtained intervention effect estimates were comparable for all three models indicates that the results reported in [4] are not very sensitive to the model-specific assumptions about missingness. Although this is good news for this specific clinical trial, results in other situations could be less robust for the missing data assumptions. Therefore, our advice is always to perform a sensitivity analysis when dealing with missing data.

Missing data is a complicated problem, which brings the need for complex statistical methods. The problem of missing data also requires (clinical) expertise on the underlying reasons for the missing data. Together, the statistician and the clinician should make decisions about which (model) assumptions are appropriate for the data at hand.

In this paper, we treated open-label medication use, following progression to dementia, and dropout as competing events. However, formally, these events are semi-competing as individuals can still progress to dementia after they drop out, or their dropout might be related to their progression to dementia. Similarly, as subjects who progressed to dementia are allowed to stay in the trial while using an open-label medication, they could formally still drop out. Another aspect is that, although different types of study dropouts were reported, we have chosen to combine all types of dropouts into one category, implicitly assuming the same missing data mechanisms for these different types of dropouts.

### Limitations

In this paper, we used a competing risk joint model to deal with the missing data. Although such a method can still provide unbiased estimates when there are different, potentially outcome-dependent causes of missing data, it also comes with extra challenges and modeling choices. Compared to mixed models, which are the traditional statistical models for longitudinal data, competing risk joint models require the specification of the survival sub-models and the choice of an appropriate association structure. Misspecification in any part of the model could affect the (accuracy of the) derived estimates. In particular, the association structure’s choice is not always self-evident or challenging to make a priori, while it may substantially influence the derived results.

## Conclusion

Missing data is a common problem in follow-up studies. Based on the observed data alone, it is impossible to tell how the missingness is associated with the observed and unobserved data. Therefore, it is important to carefully explore the effect of departures from the assumptions about missingness made in the main analysis by performing sensitivity analyses. In this paper, we have shown how one could perform such a sensitivity analysis using joint models and competing risk joint models.

### Supplementary Information

**Additional file 1: Supplementary Table 1.** Baseline characteristics for subjects in Missing group 1, Missing Group 2, and the completers. **Supplementary Table 2.** Number of subjects, observations, and events in the competing risk joint model for each outcome. **Supplementary Table 3.** Competing risk joint model results for the longitudinal sub-model using the rate of change to model the association. **Supplementary Figure 1.** Subject-specific random slopes as estimated from the mixed model and the competing risk joint model. **Supplementary Table 4.** Results of the mixed model, joint model, and competing risk model for the first type of data manipulation. **Supplementary Figure 2.** Residuals versus Fitted values for each outcome.

## Data Availability

The data are proprietary information of the LipiDiDiet clinical study group.

## Abbreviations

AD: Alzheimer’s disease
CDR-SB: Clinical Dementia Rating Sum of Boxes
MAR: Missing at random
MNAR: Missing not at random
MMSE: Mini-Mental State Examination
NTB: Neuropsychological Test Battery
