# Correction: A competing risk joint model for dealing with different types of missing data in an intervention trial in prodromal Alzheimer’s disease

**DOI:** 10.1186/s13195-023-01290-x

**Published:** 2023-10-30

**Authors:** Floor M. van Oudenhoven, Sophie H. N. Swinkels, Hilkka Soininen, Miia Kivipelto, Tobias Hartmann, Dimitris Rizopoulos, on behalf of the LipiDiDiet clinical study group

**Affiliations:** 1 Department of Biostatistics, Erasmus Medical Center, PO Box 2040, 3000 Rotterdam, CA the Netherlands; 2 Danone Nutricia Research, Uppsalalaan 12, 3584 CT Utrecht, The Netherlands; 3 Department of Neurology, Institute of Clinical Medicine, University of Eastern Finland, PO Box 1627, 70211 Kuopio, Finland; 4 Neurocenter, Department of Neurology, Kuopio University Hospital, PO Box 100, 70029 Kuopio, Finland; 5 Division of Clinical Geriatrics, Department of Neurobiology, Care Sciences and Society, Karolinska Institute, 14157 Huddinge, Sweden; 6 Clinical Trials Unit, Theme Aging, Karolinska University Hospital, 14152 Huddinge, Sweden; 7 Institute of Public Health and Clinical Nutrition, University of Eastern Finland, P.O. Box 1627, 70211 Kuopio, Finland; 8 Ageing Epidemiology Research Unit, School of Public Health, Imperial College London, St Dunstan’s Road, London, UK; 9 Deutsches Institut Für Demenz Prävention (DIDP), Medical Faculty, Saarland University, Kirrbergerstraße, 66421 Homburg, Germany; 10 Department of Experimental Neurology, Saarland University, Kirrbergerstraße, 66421 Homburg, Germany

**Correction: Alz Res Ther 13, 63 (2021)**

**https://doi.org/10.1186/s13195-021-00801-y**

Following publication of the original article [1], the authors corrected a typographical error from the editing process. The sentence “However, formally, these events are semi-competing as…” was modified for clarity. Please check if correct and if the intended meaning is retained. Otherwise, amend if deemed necessary. Yes, the sentence is correct.“ should be removed from Discussion section.

The original article [1] has been updated.
