# Supplementary material for: A competing risk joint model for dealing with different types of missing data in an intervention trial in prodromal Alzheimer’s disease
Source: Alzheimers Res Ther. 2021 Mar 22;13:63. doi: 10.1186/s13195-021-00801-y (PMC7983401; doi:10.1186/s13195-021-00801-y)

Supplementary Table 1 Baseline characteristics for subjects in Missing group 1, Missing Group 2, and the completers

|  | | **Missing Group 1 (n=98)** | **Missing Group 2**  **(n=129)** |  | **Completers**  **(n=81)** |
| --- | --- | --- | --- | --- | --- |
| Age (years) |  | |  |  |  |
| Mean (SD) | 71.3 (6.5) | | 70.9 (6.6) |  | 71 (6.9) |
| Median (min - max) | 72 (50 - 86) | | 71 (52 - 85) |  | 71 (54 - 85) |
| Sex, No. (%) |  | |  |  |  |
| Men | 49 (50%) | | 59 (46%) |  | 44 (54%) |
| Women | 49 (50%) | | 70 (54%) |  | 37 (46%) |
| Ethnic origin, No. (%) |  | |  |  |  |
| Caucasian | 98 (100%) | | 128 (99%) |  | 80 (99%) |
| Black | 0 (0%) | | 0 (0%) |  | 1 (1%) |
| Other | 0 (0%) | | 1 (1%) |  | 0 (0%) |
| Education (years) | 10.9 (4.0) | | 10.2 (3.5) |  | 10.9 (3.8) |
| Mini-Mental State Examination | 26.3 (2.0) | | 26.5 (2.0) |  | 27.4 (1.8) |
| APOE ɛ4 genotype, n (%) |  | |  |  |  |
| Carrier | 68 (69%) | | 61 (47%) |  | 43 (53%) |
| Non-carrier | 21 (21%) | | 54 (42%) |  | 32 (40%) |
| Missing | 9 (9%) | | 14 (11%) |  | 6 (7%) |
| Cognitive measures (composite Z score) |  | |  |  |  |
| NTB 5-item | -0.29 (0.58) [98] | | 0.01 (0.75) [129] |  | 0.31 (0.59) [81] |
| NTB memory domain | -0.31 (0.71) [97] | | 0.03 (0.92) [129] |  | 0.35 (0.70) [80] |
| NTB executive function | −0.13 (0.68) [96] | | -0.04 (0.74) [129] |  | 0.23 (0.66) [79] |
| NTB total | −0.22 (0.46) [97] | | 0.00 (0.61) [129] |  | 0.27 (0.50) [80] |
| CDR-SB (score) | 2.33 (1.17) [90] | | 1.82 (1.12) [117] |  | 1.12 (0.84) [74] |
| MRI brain volumes (cm^3^)‡ |  | |  |  |  |
| Total hippocampal volume | 5.21 (0.94) [69] | | 5.78 (1.23) [80] |  | 6.00 (1.21) [66] |
| Whole brain volume | 1351.00 (64.03) [59] | | 1364.77 (80.85) [74] |  | 1415.05 (89.48) [55] |
| Ventricular volume | 60.71 (28.37) [69] | | 57.55 (25.38) [102] |  | 49.10 (23.42) [62] |

Data are mean (SD) or mean (SD) [N] unless stated otherwise.

Baseline characteristics per group for subjects with baseline measurements eligible for efficacy analysis of the primary endpoint

Supplementary Table 2 Number of subjects, observations, and events in the competing risk joint model for each outcome

|  | **Control n = 158** | | | | **Active n = 153** | | | | | |
| --- | --- | --- | --- | --- | --- | --- | --- | --- | --- | --- |
| Longitudinal outcome | | *n* | N | Events *Group 1* | | Events  *Group2* | *n* | N | Events *Group1* | Events *Group2* |
| NTB 5-item composite (Z score) | | 157 | 553 | 49 | | 72 | 151 | 532 | 49 | 57 |
| NTB memory domain (Z score) | | 157 | 552 | 49 | | 72 | 151 | 530 | 48 | 58 |
| NTB executive function domain (Z score) | | 157 | 552 | 49 | | 72 | 151 | 528 | 49 | 57 |
| NTB total (Z score) | | 157 | 551 | 49 | | 72 | 151 | 530 | 49 | 57 |
| CDR-SB (Z score) | | 153 | 385 | 47 | | 75 | 143 | 363 | 47 | 57 |
| MRI total hippocampal volume (Z score) | | 121 | 325 | 37 | | 56 | 111 | 303 | 36 | 34 |
| MRI whole brain volume (Z score) | | 113 | 288 | 34 | | 51 | 102 | 269 | 29 | 32 |
| MRI ventricular volume (Z score) | | 135 | 331 | 37 | | 73 | 127 | 304 | 39 | 54 |

Supplementary Table 3 Competing risk joint model results for the longitudinal sub-model using the rate of change to model the association

|  |  | | | |
| --- | --- | --- | --- | --- |
| Longitudinal outcome | | β_3_^*^ (95 % CI) | pVal | Intervention effect over 36 months (95 % CI) |
| NTB 5-item composite (Z score) | | - | - | - |
| NTB memory domain (Z score) | | 0.068 (-0.005 to 0.142) | 0.067 | 0.205 (-0.015 to 0.425) |
| NTB executive function domain (Z score) | | -0.008 (-0.065 to 0.049) | 0.782 | -0.024 (-0.195 to 0.147) |
| NTB total (Z score) | | 0.033 (-0.011 to 0.078) | 0.141 | 0.100 (-0.033 to 0.233) |
| CDR-SB (score) | | -0.293 (-0.467 to -0.119) | 0.001 | -0.879 (-1.400 to -0.358) |
| MRI total hippocampal volume (cm^3^) | | 0.058 (0.013 to 0.104) | 0.013 | 0.175 (0.038 to 0.313) |
| MRI whole brain volume (cm^3^) | | 3.622 (0.631 to 6.612) | 0.012 | 10.865 (1.893 to 19.836) |
| MRI ventricular volume (cm^3^) | | -0.231 (-0.906 to 0.444) | 0.502 | -0.694 (-2.719 to 1.332) |

*β_3_* denotes the yearly intervention effect. Except for CDR-SB and MRI ventricular volume, higher scores indicate better performance. For NTB 5-item composite, the competing risk joint model using the rate of change to model the association did not converge.

Supplementary Figure 1 Subject-specific random slopes as estimated from the mixed model and the competing risk joint model


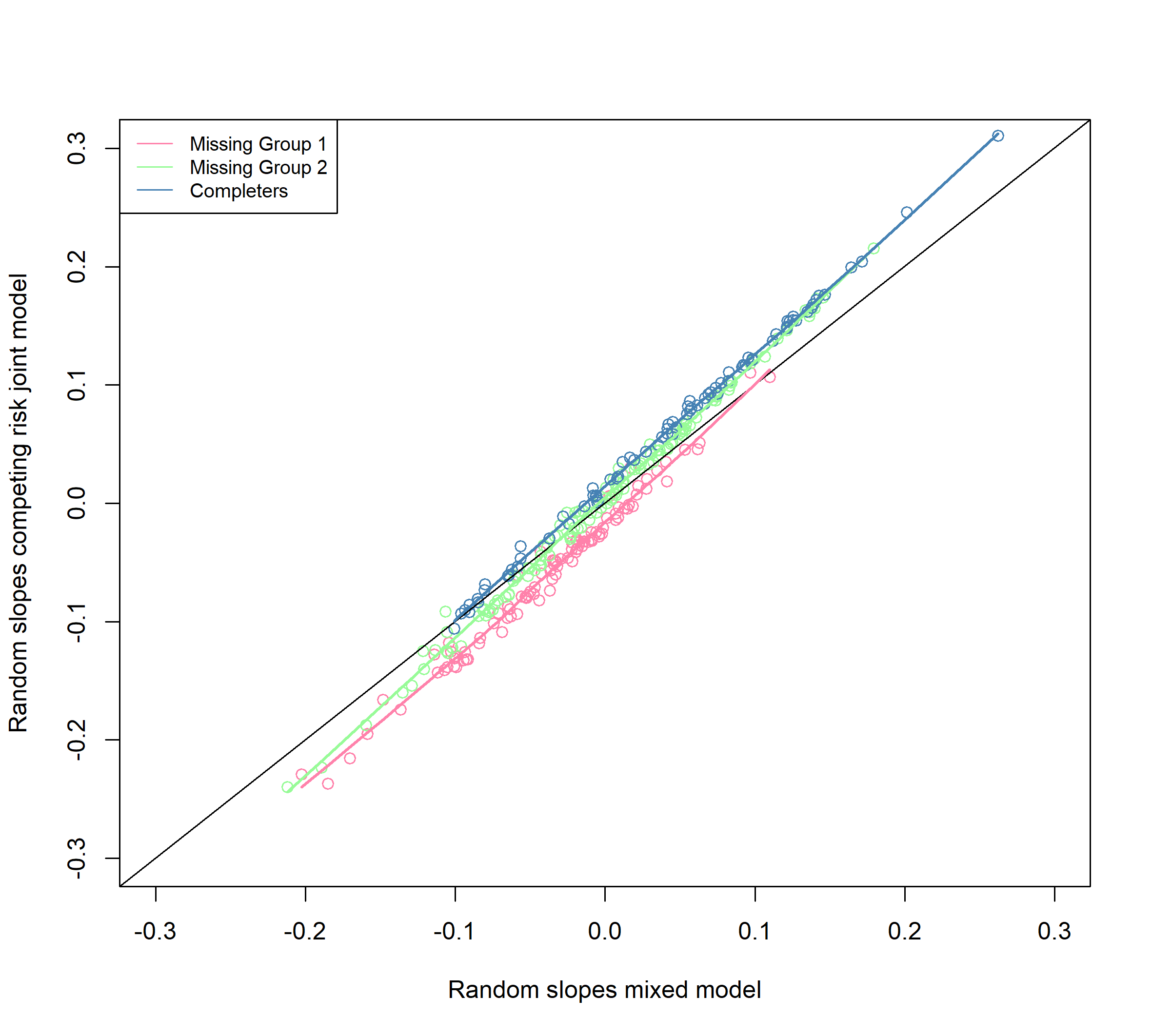


This figure compares the subject-specific random slopes for the 3 different types of subjects as estimated from the mixed and competing risk joint model with NTB memory domain as the longitudinal outcome. The black line is shown to compare the random slopes as estimated from the mixed model and competing risk joint model; values above the line indicate that the estimated random slopes are higher for the competing risk joint model, values below the line indicate that the estimated random slopes are higher for the mixed model. The competing risk joint model can distinguish between the 3 different types of subjects. As can be seen, the competing risk joint model estimates slightly higher (better) random slopes for individuals in the completer group and slightly lower (worse) random slopes for individuals in Missing Group 1 as compared to the mixed model.

For the sole purpose of illustrating the potential bias that could be introduced by ignoring the MNAR missingness and the gain in efficiency that can be achieved by modeling the different types of missingness, we manipulated the data in two different ways. For both data manipulations, we used the NTB 5-item composite outcome.

For the first type of data manipulation, we artificially created one type of MNAR missingness. We calculated the change in the NTB 5-item composite score between the first and last observation for all patients in the completer group. Then, we deleted the last (36 months) observation for the 18 subjects of the *control* group who *decreased* the most. Thus, in total, we deleted 18 longitudinal observations (1.66% of the available data). For the subjects with the extra missing observations, we set the new dropout timing to the time of what became their last available observation. For these subjects, the dropout timing was therefore artificially preponed. As a result, these subjects no longer belonged to the completer group, but we moved them to Missing Group 1 instead.

For the second type of data manipulation, we artificially created two types of MNAR missingness. We calculated the change in the NTB 5-item composite score between the first and last observation for all patients in the completer group. First, we deleted the last (36 months) observation for the 9 subjects of the *control* group who *decreased* the most. We set the dropout timing to the time of what became the last available observation for these subjects, and we moved them to Missing Group 1. This type of missingness is similar to the above, with as only difference that we deleted 9 longitudinal observations this time. Secondly, we deleted the last (36 months) observation for the 9 subjects of the *treatment* group who *increased* the most. We set the dropout timing to the time of what became the last available observation for these subjects, and we moved them to Missing Group 2. Thus, in total, we also deleted 18 longitudinal observations (1.66% of the available data).

Supplementary Table 4 shows the mixed model, the joint model, and the competing risk joint model results for the first type of data manipulation. As can be seen, in this situation, the mixed and (competing risk) joint model yield (slightly) different coefficients because they have different ways of handling the missing data. However, more apparent are the differences in the confidence intervals, and therefore p-values, illustrating the gain in efficiency brought by the (competing risk) joint models. Supplementary Table 5 shows the results for the second type of data manipulation. We observe almost identical results for the mixed and joint model but different and much more efficient results for the competing risk joint model. This is not surprising, as we created two opposite types of MNAR. With any type of missingness as event, the joint model cannot distinguish between the two types of MNAR missingness, while the competing risk joint model can.

Supplementary Table 4 Results of the mixed model, joint model, and competing risk model for the first type of data manipulation

|  |  | | | |
| --- | --- | --- | --- | --- |
| Manipulated NTB 5-item composite | | β_3_^*^ (95 % CI) | pVal | Intervention effect over 36 months (95 % CI) |
| Mixed model | | 0.031 (-0.027 to 0.089) | 0.302 | 0.092 (-0.082 to 0.266) |
| Joint model | | 0.036 (0.001 to 0.071) | 0.044 | 0.108 (0.003 to 0.213) |
| Competing risk joint model | | 0.038 (0.003 to 0.073) | 0.035 | 0.113 (0.008 to 0.218) |

*β_3_* denotes the yearly intervention effect.

Supplementary Table 5 Results of the mixed model, joint model, and competing risk model for the second type of data manipulation

|  |  | | | |
| --- | --- | --- | --- | --- |
| Manipulated NTB 5-item composite | | β_3_^*^ (95 % CI) | pVal | Intervention effect over 36 months (95 % CI) |
| Mixed model | | 0.028 (-0.028 to 0.084) | 0.327 | 0.084 (-0.084 to 0.251) |
| Joint model | | 0.028 (-0.028 to 0.085) | 0.326 | 0.085 (-0.085 to 0.255) |
| Competing risk joint model | | 0.035 (0.003 to 0.066) | 0.030 | 0.105 (0.010 to 0.199) |

*β_3_* denotes the yearly intervention effect.

Supplementary Figure 2 Residuals versus Fitted values for each outcome


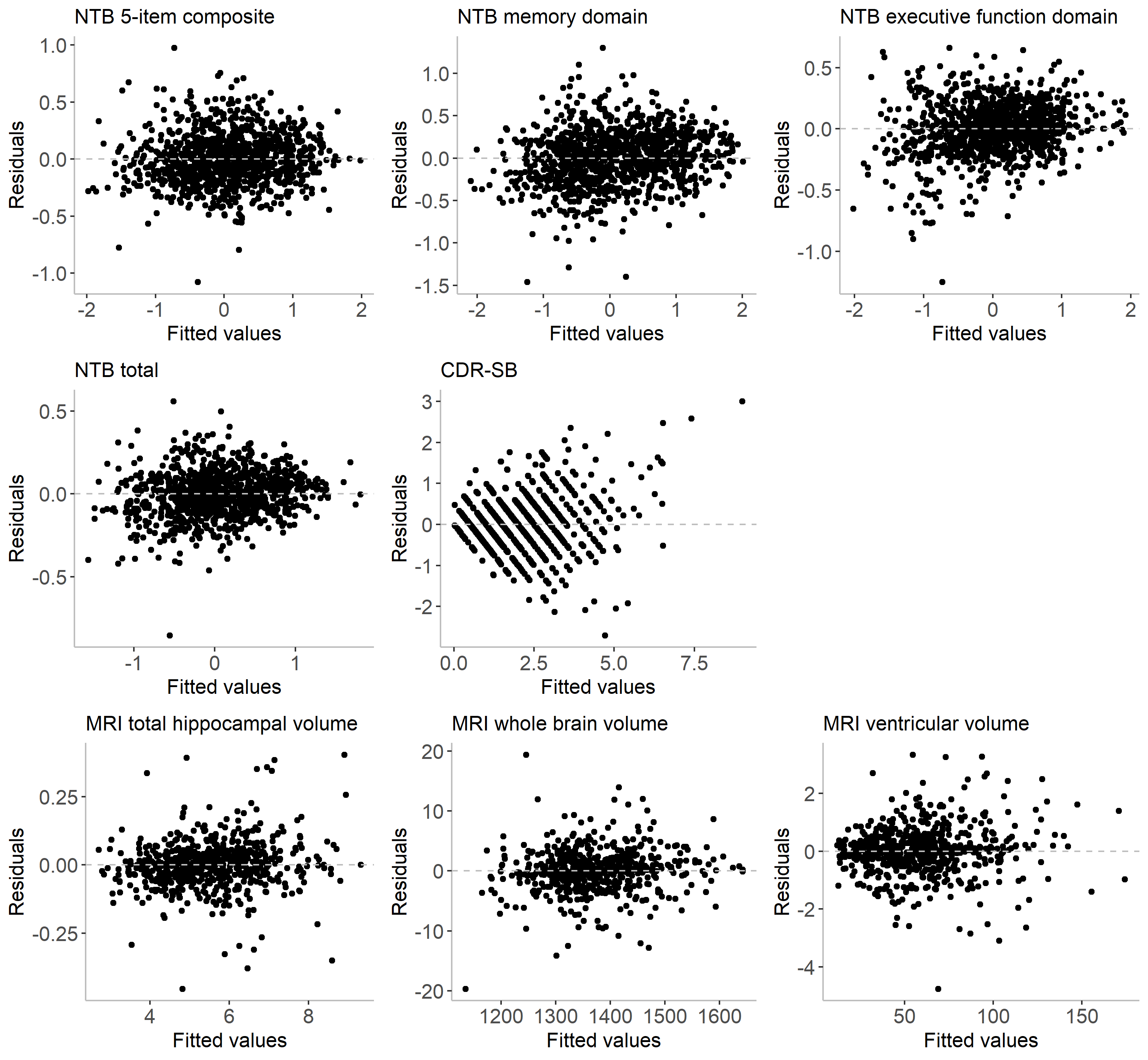

Supplement: Supplementary file 1 — Additional file 1: Supplementary Table 1. Baseline characteristics for subjects in Missing group 1, Missing Group 2, and the completers. Supplementary Table 2. Number of subjects, observations, and events in the competing risk joint model for each outcome. Supplementary Table 3. Competing risk joint model results for the longitudinal sub-model using the rate of change to model the association. Supplementary Figure 1. Subject-specific random slopes as estimated from the mixed model and the competing risk joint model. Supplementary Table 4. Results of the mixed model, joint model, and competing risk model for the first type of data manipulation. Supplementary Figure 2. Residuals versus Fitted values for each outcome. [file 13195_2021_801_MOESM1_ESM.docx]
